# On the Role of Speed in Technological and Biological Information Transfer for Computations

**DOI:** 10.1007/s10441-022-09450-6

**Published:** 2022-10-26

**Authors:** János Végh, Ádám József Berki

**Affiliations:** 1 Kalimános BT, Debrecen, 4032 Hungary; 2 Department of Neurology, Semmelweis University, 1085 Budapest, Hungary; 3 János Szentágothai Doctoral School of Neurosciences, Semmelweis University, 1085 Budapest, Hungary

**Keywords:** Computing paradigm, Technological computing, Biological computing, Information transfer speed, Information storage, Lifelong learning, Redundancy, Temporal behavior, Machine learning

## Abstract

In all kinds of implementations of computing, whether technological or biological, some material carrier for the information exists, so in real-world implementations, the propagation speed of information cannot exceed the speed of its carrier. Because of this limitation, one must also consider the transfer time between computing units for any implementation. We need a different mathematical method to consider this limitation: classic mathematics can only describe infinitely fast and small computing system implementations. The difference between mathematical handling methods leads to different descriptions of the computing features of the systems. The proposed handling also explains why biological implementations can have lifelong learning and technological ones cannot. Our conclusion about learning matches published experimental evidence, both in biological and technological computing.

## Introduction

The computing model proposed by von Neumann in his famous "First Draft" (von Neumann 1993), is *bio-inspired*, despite the common fallacies: he discussed the computing process implemented in both technological (vacuum tubes) and biological (neurons) computing systems. However, because von Neumann made omissions *validated only for [the timing relations of] vacuum tubes*, his *simplified classic paradigm* cannot be applied to other technologies (Williams 1993; Godfrey and Hendry 1993). Because of the omission that we can neglect the transfer time apart from the computing time, implementations based on the classic paradigm are not *biology-mimicking* ones. Von Neumann, of course, could not foresee the dawn of modern computing technologies, but he warned that *computing paradigm must be revised when technology changes* and that it would be *unsound* (sic) to apply his *simplified paradigm* (not to be confused with his *model of computation*!) to neural computing. Given that he outlined that his proposal was about *the logical structure* of a computing implementation, it is not a very usable classification criterion whether (the otherwise undefined) von Neumann *architecture* is biomorphic (for a review see Schuman et al. 2017) or not.

## Computing Paradigms

No doubt that von Neumann’s model is valid for all kinds of computations: the operand(s) (in the form of some physical carrier) must be delivered to the operating unit where the computation takes place. The computation cannot even start (as pointed out by von Neumann (1993)) until the operands are completely delivered to the input section of the computing unit, and similarly, transferring the result cannot even begin until the computation is completed and the result is available in its output section. Because of this, the *operand transfer* and *computing* mutually block each other: some "idle" activity is inherently present in the *computing process*.

In the computing model, one must consider both the time needed to transfer the operand and the time to make computations with it and the presence of blocking constraint. To calculate the *total time of a computing process* is not simple at all: it depends on both hardware characteristics and workload type, so some neglections must be made. Because of these difficulties, *the classic paradigm neglected the transfer time, so the blocking constraint in the classical paradigm means only logical dependence*. Technological computing is based on the Hardware-Software contract (Asanovic 2009): mathematics provides the solid theoretical basis for computing but neglects the data transfer time, and technology must adapt itself to the interface defined by von Neumann a three-quarter century ago, and for [the timing relations of] vacuum tubes only.

We surely know that the simplified model is not valid for our current technology. As the technology develops, it becomes evident that the classic paradigm cannot describe real-world implementations, neither technological (electronic) nor biological (neural) ones. Furthermore, in technology, it leads directly (Végh 2021) to the idea of unlimited computing capacity and workload-independent processing time. In electronics, mainly the issues experienced in connection with building so-called neuromorphic computers led the researchers to the idea that "*More physics and materials are needed. Present-day electronics are not enough*" (Markovic et al. 2020). We can add: *Present-day computing science is not enough: more physics in theory is needed.*

However, computing science does not want to admit that the physical implementations of computing must also include physics, despite the existence of the ready-made mathematics (Minkowski 1908). In some sense, hundred years after inventing the Minkowski-mathematics, it is still a scandal (Walter 2008) to consider that *the theory of technological implementation of computing must also include some modern physics*. The important consequences include (but are not limited to) inefficient processor chips (Hameed et al. 2010), enormous power consumption (Waser 2012), the experience of "dark silicon" (Esmaeilzadeh et al. 2012), the stalled supercomputer performance (Végh 2020; Simon 2014), the stalled Artificial Intelligence (AI) development (Hutson 2020; Végh 2021) and failed brain simulation (Abbott 2020). The at that time "*disciplinary analysis of the reception of Minkowski’s Cologne lecture reveals an overwhelmingly positive response on the part of mathematicians and a decidedly mixed reaction on the part of physicists*" (Walter 2008) has turned to its exact opposite. The description is generally accepted in physics (and resulted in the birth of a series of modern science disciplines) but completely refused in mathematics-based computing science.

In biology, it was evident that the transfer (conduction) time must be considered together with the computing (synaptic) time (in this sense, presynaptic to postsynaptic transmission time). The name "spatiotemporal" and a (separated) time dependence is commonly used (Maass et al. 2002), in the sense that Precise Firing Sequence (PFS) "*tended to be correlated with the animal’s behavior*"; furthermore, that "*the results suggest that relevant information is carried by the fine temporal structure of cortical activity*" (Prut 1998). The "*neural dynamics*" was studied and "*spatiotemporal spreading of population activity was mapped*" (Plenz and Aertsen 1996) by methods used to describe the *static computing methods*: interspike intervals histograms, auto-correlation and cross-correlation. Because of the peculiarities of this information handling, *there are severe doubts whether the notions of the classic neural information theory are valid for biological computing systems* (Végh and Berki 2022). The correct method of describing biological computation is still missing, given that the significant item of the computing is missed: *the time and position are connected through the information transfer speed* (called conduction velocity).

## The Effect of the Finite Speed of the Information Carrier

Transfer time is neglected apart from computing time when using the classic computing paradigm. In other words, "instant interaction" (infinitely large transfer speed, or in other words, infinitely small physical computing system size) is assumed. However, in all physical implementations, the information has some material carrier: inertial mass, electromagnetic or gravitational waves, electrons/ions, neurotransmitters, etc. The kind of the carrier and its transfer mechanism limit the speed of the information transfer, so the physical size of the computing system matters.

In electronic technology, the transfer speed is $10^8$ m/s; in biology (speed of neural transfer), it is $10^1$ to $10^2$ m/s; that is, the speed of electronic signals is several million times higher than the speed of neural transfer. At dozens of centimeters physical size, the transfer time is also several million times lower than in neural systems (such as our brain). Because of this difference, in biological computing systems, a "spatiotemporal description" was assumed from the beginning when studying neural operation. In contrast, in (electronic) technological computing systems, the "instant interaction" initially seemed to be a good approximation. However, we have good reasons to introduce a finite interaction speed in science and computing technology (Végh 2020).

Initially, both technological and biological *computing* worked on the same *msec* time scale (Eckert and Mauchly 1945). In contrast, the information *transfer* speed was several million times higher for technological computing than for the biological one. This difference made the classic paradigm a reasonable approximation for technological computing. However, the evolution of technological computing systems quickly invalidated the assumption of the classic paradigm: the processing time moved from the several *msec* region to the sub-nanosecond region, significantly increasing the transfer-to-computing time ratio (Simon 2014). Although the *component density* of processors followed Moore’s observation for decades, the propagation speed of the material carrier, the electromagnetic interaction, remained the same. Similarly, the physical size of the technological computing systems remained several dozens of centimeters. Because of these changes, in technological computing systems the *transfer time* remained the same, while their *computing time* became millions of times shorter (Luk 2019). The stealthy nature of technological development resulted in that in current technological computing, the transfer time is not only not negligible apart from the computing time, but even it is longer than that. The timing relations of biological computing did not change, so this subtlety of the technological evolution resulted in the timing relations of current computers being much closer to those of our brain than those assumed in the abstract model. According to the inventor of the classic paradigm, it is *unsound* (sic) to use his simplified paradigm for the timing relations of our current technological computing system.

Given that a physical carrier delivers the information (in all implementations, although the representation of the information is different (Végh and Berki 2022)), the transfer time is crucial in all computing implementations. As the computing model requires, the operand must reach the operator unit’s input section, and for all physical carriers, the transfer speed is finite. Whether one can neglect the transfer time apart from the processing time depends on the implementation technology.

**Fig. 1:**
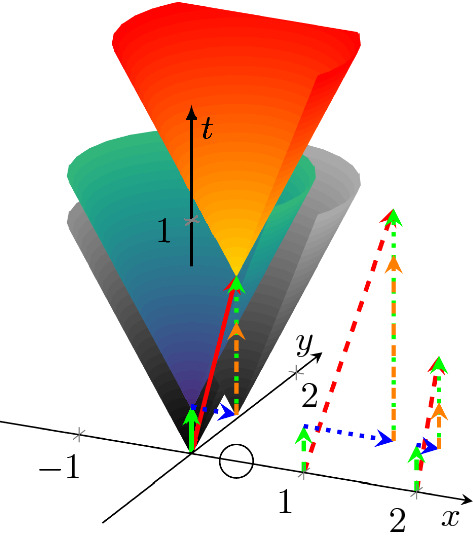
The temporal diagram, i.e., the way of calculation to combine the spatial distance (transmission time, blue arrows) and computing time (green arrows) illustrated in the time-space coordinate system. The orange-green vertical arrow shows that the second computing unit must idly wait until the transmitted result reaches its position, because of the finite transmission time. The axes *x* and *y* refer to space coordinates (transformed to time using the conduction velocity), the axis *t* refers to the time itself. The arrows starting from points 0, 1 and 2 on the *x* axis illustrate timing for three different propagation speeds. The red vector points from the beginning to the end of the process. Its length may serve as a statistical entity to describe temporal distance of the units. (Color figure online)

A somewhat simplified view of the abstract computing process is shown in Fig. 1. Here we have processing units (such as processor cores, logic gates or neurons) at different positions of an (*x*, *y*) plane which are performing operations at different times shown on the time axis *t*, perpendicular to the (*x*, *y*) plane. For the exact interpretation of the coordinate system and math details see (Végh 2020; Végh et al. 2021). The information must be transferred between places ($x_1,y_1$) and ($x_2,y_2$), which requires *transfer* time. After that, the computation can be done at the place ($x_2,y_2$), which takes *computing* time. The known speed of information transfer enables us to calculate the distance in time units: the speed of the information carrier interconnects the temporal and spatial distances. Minkowski (1908) elaborated on the exact form of that dependence; however, we use a different scale factor. To use the same unit for the four dimensions, in our time-space system we rescale the spatial coordinates to time by dividing the distance values by the conduction velocity, while in the famous case of the space-time system (the theory of special relativity) the time is rescaled by multiplying it by the speed of light.

In Fig. 1 (as well as in all similar figures), all coordinates represent time but labeled as coordinates *x* and *y*. The spatial length of transfer operations is represented by horizontal (blue) arrows and the temporal length of computing operations by vertical (green) arrows. The time of an action is provided by the *t* coordinate of the vertical arrows. However, the result is produced at a place from where additional time is needed to arrive at the next processing unit.

The figure shows the dramatic difference between the discussed paradigms: according to the classic (time-unaware) paradigm, the length of the blue vectors (the time needed to transfer information to the other point) is zero, that is, the length of the green vectors (*computing tim*e) equals to the length of the red vector. In other words, the classic paradigm assumes that all computing objects are in the origin, and no time is needed to access them.

## The Effect of Synchronization

According to von Neumann’s model, there is an implementation-independent need to synchronize the operations data transfer and computation to each other (for a detailed discussion, see Végh (2021)), so the implementation must provide means to keep its operations synchronized. Different means are provided in various implementations, leading to drastically different features.

In biological implementations, source neurons fire (send information) when they need to, and target neurons receive it after the charges arrive via their chemical and/or (less common) electrical synapses (Pereda 2014). Sending and receiving spikes involves many complex processes which must follow each other and contribute to the time of the synaptic transfer.

The arrival of the spike in the presynaptic component, the nerve terminal (i.e. charge distribution in the input/sender) triggers the release of neurotransmitter chemicals into the synaptic cleft. There is no direct signal charge transfer from the terminal to the receiver (even if some transmitter molecules carry charge). The transmitters having diffused close enough to the receiver get bound by receptors in the postsynaptic component (receiver) resulting in a change in the membrane conductance and charging of the membrane by ions from the extracellular space. There are also relevant dynamic mechanisms involved in changing the quantity, probability and timing of the charge induced in the receiver membrane dependent on its previous experience or the presence of neuroactive modulators.

The appearance of charge on the postsynaptic membrane (i.e., the end of transfer time) triggers computing (i.e., marks the beginning of computing time). Similarly, firing (when the threshold potential is reached) marks both the end of the computing time and the beginning of the data delivery time. All this machinery works in the individual neurons independently.

That is, *the arrival of a spike is a synchronization signal*^1^ as well: the zero time of the synaptic conductance function *gsyn*(*t*) (Koch 1999) is set to the arrival of the spike. The stalled ions create a potential that opens the potential-controlled ion channel (formulated as “*Synaptic inputs effectively open ’holes’ in the membrane*” (Koch and Poggio 1983)). Notice that while receiving a spike, the conductance *gsyn*(*t*) of the receiver synapse changes: after reaching its peak conductance, it decreases again to zero. This change limits the amount of charge delivered to the membrane (that is, the charge distribution in the received spike and the one reaching the membrane are different). As the spikes keep arriving, the charge continuously reaches the membrane. It increases its potential in an analog way.^2^ In this way *the length of the computing time* (until the membrane potential reaches its threshold value) *depends on the amount of the charge, its arrival time and its arrival speed*. After the membrane reaches its threshold potential, the computing time ends, and the firing period begins. However, it takes time (the duration of the spike, see ’Signal delivery’ in Végh (2021)) until the signal reaches the output section of the neuron: the membrane provides an ’End computing’ (Végh 2021) signal, and $\approx 200~ \mu s$ later (Singh et al. 2021) the spike begins. *This mechanism provides the auto-synchronization of biological computing.* This aspect is consequently neglected in spiking neural networks’ technological implementations; for a review, see (Schliebs and Kasabov 2013).

**Fig. 2:**
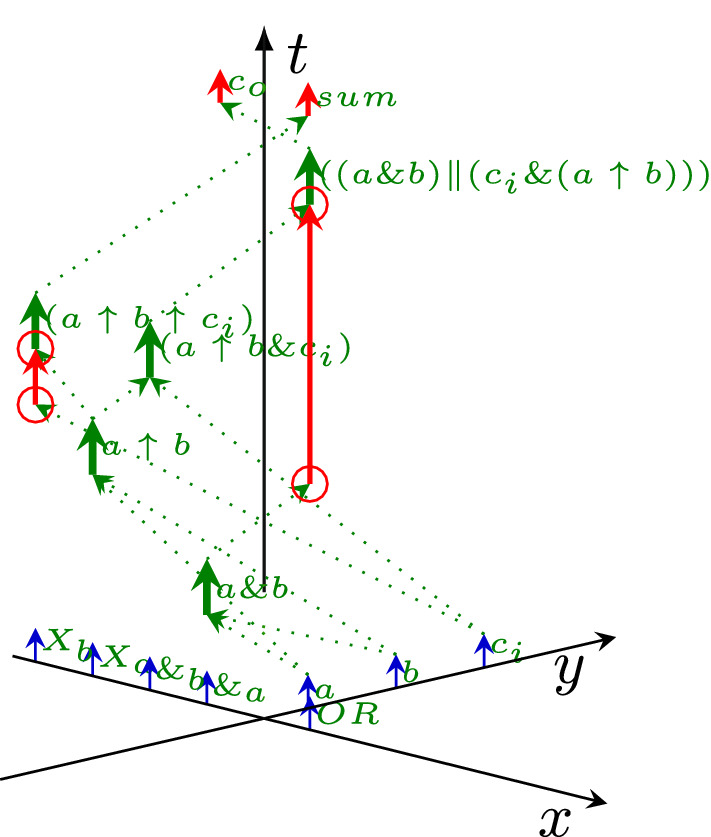
The operation of a technological one-bit adder, with "pointless" synchronization (red circles), see Listing 1. The input signals *a*, *b* and $c_i$ are aligned along axis *y* (the input section), the computation takes part in gates aligned along axis *x*, and the output signals $c_o$ and *sum* aligned again along axis *y* (the output section). The figure uses the coordinate system introduced in Fig. 1

In contrast, due to the lack of a start signal in technological implementations, a gate (or another digital object) starts to "compute" its result as soon as any of its inputs changes. It provides the corresponding output after its *fixed length computing time* passes (without providing signal ‘End computing’ (Végh 2021)). The gates always have an output signal. Whether this output signal corresponds to the value expected based on the functionality (AND, OR, XOR, etc.; on the figure see the operators denoted by &, $\Vert$ and up-arrow, respectively) of the gate depends on whether all operands succeeded in arriving at the corresponding input sections of the gate before the computing began. After computing, the result arrives at the output section of the unit. The lack of auto-synchronization in technological computations results in internally undefined states (and is the main reason for the inefficient processor chips (Hameed et al. 2010) and their enormous power consumption (Waser 2012)), as shown in the operating diagram of a one-bit adder in Fig. 2. The circuit works with three inputs (the two operands plus a carry bit from the previous bit) and produces two outputs (the result plus a carry bit for the next adder). The corresponding code in SystemC [33] is shown in Listing 1. Notice how the pure logical dependency, a consequence of the time-unaware paradigm (called also "von Neumann programming style" (Backus 1978)) is converted to temporal dependence by the technological implementation, and that the adder performs payload (computing) work only in the periods denoted by thick green arrows; the rest is non-payload (transfer) time. Notice that both computing and transfer times are partly parallel (overlapping).
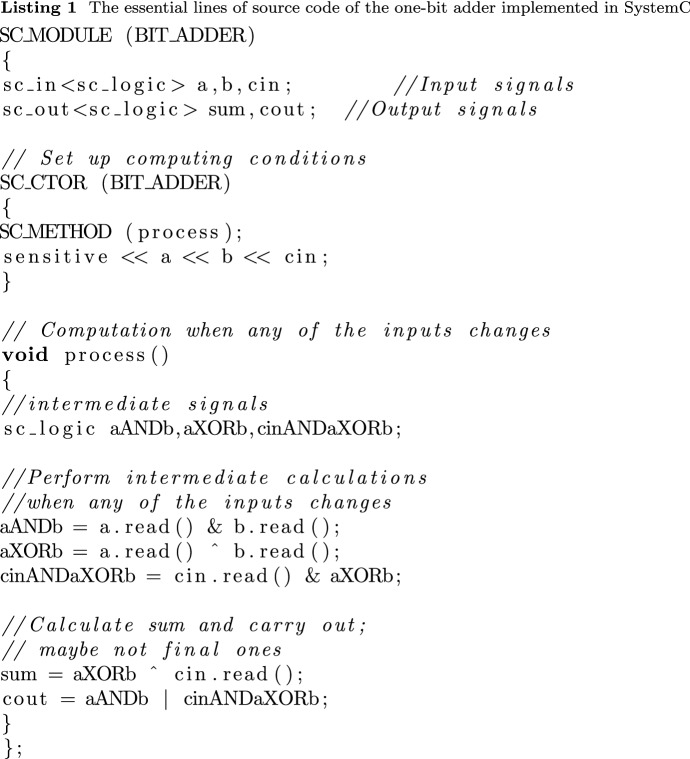
 At the red circles at the bottom of the red arrows, one operand (the result of a previous computation) arrives at its target gate (green arrowhead in the red circle), and (after the computing time) it may change the state of the target gate (depending on its previous state). However, the state *may be or may not be* the final result of the operation: the second operand is still missing (although the input section has a well-defined signal level). After some time (the red arrowhead in the upper red circles), the second operand also arrives, and it may change the state of the target gate (depending on its previous state). In the time fraction corresponding to the red vector, the output value of the gate is undefined, and so is the result of the one-bit addition. Also, notice that upon arrival of the second operand and performing the computation the gate represents, the signal still must arrive at the output section of the adder. Without synchronization, especially in a several-bit adder, the information available in the output section of the adder may change several times^3^ during the operation (Végh 2020).

Von Neumann emphasized the role of synchronization. His classic analysis resulted in one more important (and, for the intended vacuum-tube implementation, correct) conclusion: the initial design is significantly simplified using a central synchronization clock signal. This is why a synchronization signal is used to validate the output signal: for the external world, signal $c_o$ is only achievable after the central clock signal arrives. Given that the output signal $c_o$ is, at the same time, the input signal $c_i$ of the next one-bit adder, the clock signal must reach the first and the last one-bit adder at different times. This need anyhow results in some "skew" in the clock signal. Given that the total idle time increases with the number of bits comprised (from the beginning of the idle time in the first bit to the end of idle time in the last bit), in more extensive designs, the need for introducing several clock domains (Waser 2012) appears.

**Fig. 3:**
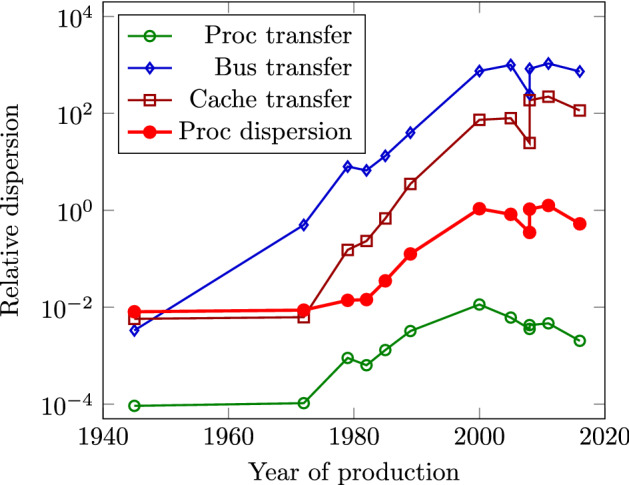
The history of different relative dispersion characteristics of processors, in function of their production year. Notice how cramming more transistors in a processor changed their temporal characteristics disadvantageously. The technological data are calculated from publicly available data (https://en.wikipedia.org/wiki/Transistor_count) and from Eckert and Mauchly (1945), as described in Végh (2021)

Von Neumann’s detailed analysis has shown that using a central synchronous signal is advantageous only when the dispersion of clock signals (how uniform are the temporal lengths of the atomic computations) is negligible. However, the evolution of technological computing quickly increased the dispersion (essentially the total to payload activity, see Fig. 3 and the detailed discussion in Végh and Berki (2020), Végh (2021, 2020)). Given that miniaturization took place only inside the processor while the physical bus size remained the same, the dispersion of bus transfer grew up unproportionally and the cache memories shined up when the dispersion of bus transfer started to differ strongly from the dispersion of the processor. It is already noticed that the advantages of using central synchronization are lost in current technological implementations. Recently, the idea of asynchronous operation was proposed (Kumar et al. 2013). *In our current technologies, the dispersion is not negligible* even within processors, and especially not in computing systems, from the several centimeter long buses in our PCs (Waser 2012) through the wafer-scale systems (Grubl et al. 2020) to the hundred meter long cables in supercomputers (Végh 2020). Today, the relative dispersion (the timing relations) of technological computing is much closer to that of biological computing than to that assumed in the classic computing paradigm.

## Time, Information Storage and Learning in Biological Implementation

**Fig. 4:**
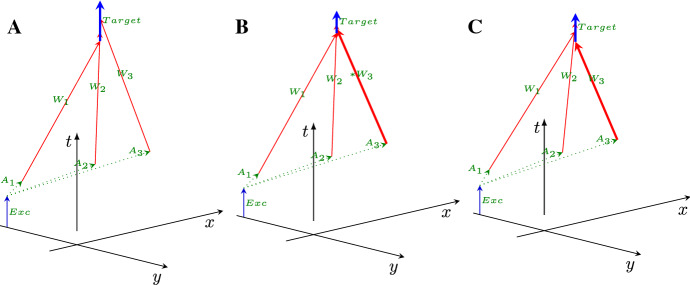
How neurons learn. **A** The initial state **B** short time learning, changing synaptic weight $W_3$ by +50% **c** long time learning, changing conduction velocity $C_3$ by +10%. The figure uses the coordinate system introduced in Fig. 1. The figure shows a neuronal assembly, where the assembly member $A_3$ changes the timing of *Target* due to changing the synaptic weight $W_3$ and conduction velocity $C_3$, respectively

In biology, it was evident from the beginning that the measurable quantities change with time and space: this experience is called "spatiotemporal". It is similarly evident that, unlike technological computing systems, the brain does not feature a unique, perfectly synchronous clock to regulate communication and computing (Antle and Silver 2015). The common experience (Koch 1999) shows that the outputs of biological neurons depend not only on their inputs (they compute with their inputs) but also on their internal state (they store information (Sterling and Laughlin 2017; Buzsáki 2019)^4^). Furthermore, biological systems can adapt to short-term and long-term changes in the external world: they can learn. Fortunately, the time-aware paradigm offers a natural explanation for those phenomena. We use "*the broad definition of learning: use present information to adjust a circuit, to improve future performance*" (Sterling and Laughlin 2017). However, the definition needs to explain also, what is the "present information" (or information at all (Végh and Berki 2022)). Figure 4 shows how the model explains in what form the information is stored and adjusted in biological neurons. Furthermore, it explains and connects those two learning modes.

Figure 4 (in the coordinate system also shown in Fig. 1) shows how biology implements short and long-term learning using a neuronal assembly. We assume that the excitation of a neuron at coordinates (−1, 0, 0) occurs, and a neuron fires to an assembly (aligned along coordinate axis *y* at *x* values −0.3, 0.1 and 0.4). The figure assumes that the excitation branches towards three assembly members *A* after passing to the branching point. The assembly members $A_i$ send spikes to their common *Target* neuron at position (−1.5, 0.7). The corresponding synaptic weights of the target neuron are $W_1, W_2, W_3$ (assumed to be equal for *A*), and three received spikes may cause the target neuron to fire (the sum of potential contributions of the three spikes is just above the threshold). Given that the position of the assembly members and their firing times slightly differ, so differ the arrival times (see the red arrowheads) of the spikes from the assembly members at the position of the target.

The *Target* is initially in rest. When the first spike arrives, it increases the potential of the membrane, and so do the second and third spikes somewhat later. With the charge delivered by the third spike, *Target* reaches its threshold, and (after some "processing time": charging the membrane) it fires. The blue arrow length denotes the length of the computation: its bottom is at the arrival time of the first spike and its head is at the beginning of the refractory period of the neuron. Given that after reaching the threshold, some time is needed to charge the membrane to its operating potential, the length of the arrow includes an extra contribution.

The total operating cycle of the neuron is given as

$$ T_{Computing} = T_{Triggering} + T_{Charging} + T_{Refractory} $$

Here we assume that the computation will not fail. $T_{Triggering}$ is the time which *Target* needs to collect potential contributions from its synapses to reach the threshold, $T_{Charging}$ is the time required to charge the membrane to its maximum potential, $T_{Refractory}$ is needed to reset the neuron to its initial state. Usually, some idle time $T_{Idle}$ (no neural input) also follows between the "computing operations", which can make $T_{Computing}$ longer. That is, the operating frequency (its firing rate, the reciprocal of $T_{Computing}$) of the cyclic operation is

1 $$\begin{aligned} F=\frac{1}{\underbrace{T_{Triggering}}_{Memory+Network}+\underbrace{T_{Charging}+T_{Refractory}}_{Biophysics}+\underbrace{T_{Idle}}_{Network+Neuron}} \end{aligned}$$

Given that $T_{Charging}$ and $T_{Refractory}$ are defined biophysically, the only way to adjust firing rate of neurons is to change the $T_{Triggering}$ and $T_{Idle}$ times. The latter is relevant only in periodic operations and relates to how long to ’pause’ between computations is enabled and how quickly the membrane of the neuron reaches its activation threshold potential. In this simplified discussion, we do not consider that between arrivals of spikes, the membrane loses some charge. That is, the (biophysically defined) maximum operating frequency is

$$ F_{Max} = \frac{1}{T_{Charging} + T_{Refractory} } $$

The time $T_{Triggering}$ can be adjusted by the neuron either by collecting more charge to its membrane from its synapses or collecting the same amount of charge in a shorter time or combining them. Biology provides mechanisms for both ways of adjusting the triggering time. The two modes differ in their implementation speeds and operating costs, enabling the biological systems to find a proper balance between the two.

It was noticed that neurons have memory (for example, "deviations from the equilibrium [membrane potential]" is mentioned as "a form of intracellular memory" (Li and Tsien 2017)). Our analysis shows that the result of a neuronal operation depends on both the inputs of the neuron and its internal memory. The neuronal memory depends on the former activity of the network. A complex convolution of the past (a weighted integral of the respective network activity in the past relative refractory period) and present network activity defines the momentary firing frequency of the neuron. We cannot understand the dynamic operation of a neuron without considering its network: the neuron is an unusual simple processor which takes its inputs from its time-dependent environment. The opposite claim is also valid: we cannot describe a neural network without accounting for the temporal behavior of its neurons. Végh and Berki (2022) provides a more detailed analysis.

In the learning process, one mechanism to collect more charge from the input spike that hits its synapse is to increase the synaptic weight $W_i$ corresponding to assembly member $A_i$. The axonal spike reaches the presynaptic terminal and the depolarisation of the membrane by the entry of positive charge leads to neurotransmitter release. Increasing $W_i$ means a larger increase in the potential of the membrane. As observed (Benke et al. 1998), "*elementary channel properties can be rapidly modified by synaptic activity*". Increasing transmitter concentration extracellularly does not necessarily lead to increased synaptic weights as in some synapses receptors are saturated by the transmitter released from a single synaptic vesicle. Increasing effect in such synapses can occur by incorporating more receptors, one of the mechanisms of synaptic plasticity, but this takes time, hence termed long-term potentiation (LTP). Another way of increasing effect is by modifying existing receptors e.g. by phosphorylation of the protein which is faster. (More concrete mechanisms are discussed in Morrison et al. (2008).)

Another mechanism is to decrease the time needed to transfer a spike from $A_i$ to *Target*: biology can wrap the axons into an insulating lipid layer to accelerate spike conduction which is provided by some of the supporting cells of the brain called oligodendroglia. Given that the transfer speed (conduction velocity) on thicker axons gets higher, the spike arrives in a shorter time and, in this way, contributes to the potential of the membrane at an earlier time (i.e., the membrane reaches its threshold potential earlier, too). This mechanism is less expensive in terms of energy consumption but needs a significantly longer implementation time. The aspect that changes in conduction velocity "*could have profound effects on neuronal network function in terms of spike-time arrival, oscillation frequency, oscillator coupling, and propagation of brain waves*" (Pajevic et al. 2014), and that "*Node of Ranvier length [can act] as a potential regulator of myelinated axon conduction speed*" (Ford et al. 2015; Arancibia-Cárcamo et al. 2017) have been noticed, but *the role of time in storing information and learning has not yet been discussed*.

Given that both mechanisms result in shorter $T_{Triggering}$ times, they cause the same effect: the computing time (or, in other words: the firing rate) changes. When the firing rate changes, one cannot tell which mechanism caused it. This equivalence is why nature can combine the two mechanisms: a neuron can increase its firing rate quickly (as a trial), and (on success, that is, if that learned condition is durable), it may decide to reimplement ("remodel" (Almeida and Lyons 2017)) its knowledge in a less expensive way. It makes the corresponding axon thicker. After that, the needed weight $W_i$, which was increased for the short-term learning (experimental) period, can be decreased to conserve the learned long-term (stable) knowledge to reduce the energy consumption. The effect (the learned knowledge) remains the same.

Our assumptions seem to be supported by anatomical evidence that "*individual anatomical parameters of myelinated axons can be tuned to optimize pathways involved in temporal processing*" and that "*the internode length decreases and the node diameter increases progressively towards the presynaptic terminal, and ...these gradations are crucial for precisely timed depolarization*" (Ford et al. 2015). However, the change of thickness of axons is measurable only after weeks or months; presumably, so is the decrease in the transfer time to the synapse. It was observed (Almeida and Lyons 2017) that "*neuronal activity can rapidly tune axonal diameter*" and "*activity-regulated myelin formation and remodeling that significantly change axonal conduction properties are most likely to occur over time-scales of days to weeks*".

This mechanism reveals that short-term and long-term learning perform the same action: they reduce the processing time using two different biological implementations. Increasing neurotransmitter concentration leads to shorter computing time, and increasing axon thickness leads to faster transmission time. As our model predicts, the processing time decreases (the firing rate increases). It means that short time and long time learning are just two sides of the same coin. Our analysis seems to underpin that it was correctly explained (Sterling and Laughlin 2017) "*we should not seek a special organ for ’information storage’ - it is stored, as it should be, in every circuit*". Mainly because "*information stored directly at a synapse can be retrieved directly*". Besides, our analysis adds the discovery that remained hidden: *the information is stored through handling time* or, in other words, *adjusting temporal processing*. Biology takes advantage of the low speed of information propagation^5^; one more evidence for the neural design principle (Sterling and Laughlin 2017): ”*Send only information that is needed, and send it as slowly as possible*”. This information handling is why biological systems have lifelong learning capability without implementing switches "Learn On/Off" and "Short/Long term".

Biological systems have a complex network of partly pre-programmed neurons with well-defined initial synaptic weights (as reflected by their firing rates). Our time-aware paradigm offers the chance to investigate and understand those phenomena quantitatively. As the elegant investigation (McKenzie et al. 2021) proved, as an implied side result,^6^ the result of learning manifests in that the affected neurons change their firing frequency using the mechanism described above. Furthermore, a way to find out directly whether it is a long or short time learning (whether the change in $T_{Triggering}$ correlates with the change in the corresponding conduction speed or the synaptic strength): the firing rate is the inverse of the governing rule, the temporal relations.

Notice that in Fig. 4A the spike from $A_3$ arrives last, immediately before *Target* fires. According to Hebb’s observation (Hebb 1949), that the synaptic weight of the synapse contributing the last spike, $W_3$, is increased in Fig. 4B by 50%; putting $A_2$ in the position of the winner for the next learning cycle. This effect provides the experienced dynamical change of features needed for life-long learning. Both wiring and redistribution of synaptic weights go through several phases of growth and reduction during development in biological systems, partly due to pre-programmed genetic programmes and partly due to environmental stressors. And if the biological system becomes resilient enough to perturbations e.g. by the end of adolescence, a stable state emerges into adulthood. The initial period represents most of short-term and long-term learning.

The temporal feature changing is also the key to understanding redundancy. As one can conclude from the experimental results in Losonczy and Magee (2006), using *simultaneous* firing, 6–7 spikes would be sufficient for charging the membrane above its threshold value. Given that the membrane discharges between the arrival times of the charge contributions from the individual spikes and their arrivals are poorly concerted, up to 20 spikes are needed in practice to produce firing. This phenomenon and the learning mechanism above explain that if one of the dominant assembly members dies out, the synaptic strength of some formerly ‘obsolete’ assembly signal can increase. After some training, the operation stabilizes using the new assembly member.

## Time, Information Storage and Learning in Technological Implementation

In technology, from the point of view of mimicking biology, the computing paradigm is the one invented a three-quarter century ago for vacuum tubes. In its simplified form, this classic paradigm (von Neumann 1993) is based on the (for vacuum tubes, valid) assumption that the transfer time can be neglected apart from the computing time. The most harmful consequence of neglecting transfer time (that is, assuming "instant interaction") is that the paradigm can be used to describe neither how information is stored nor changing the transfer speed. Technology must use drastically different implementation methods that manifest in drastically different behavior of technological and biological systems.

**Fig. 5:**
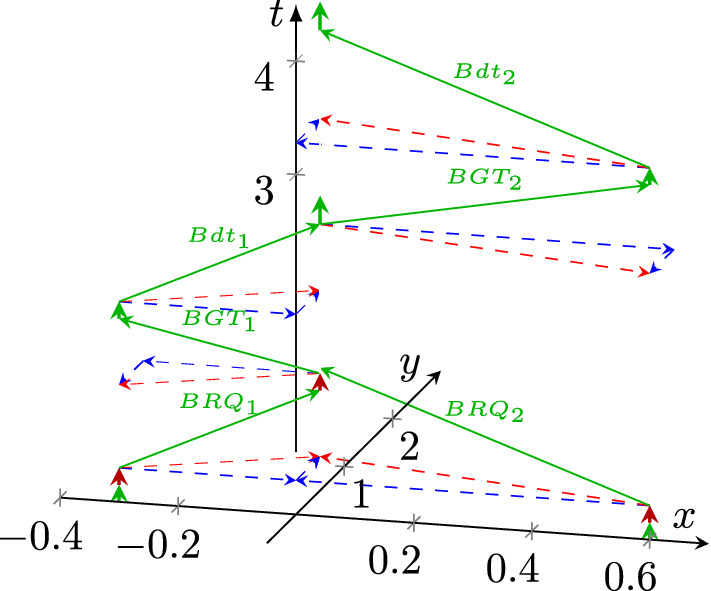
The temporal operating diagram of a technological high-speed single bus: the bus delivers data only in the fractions denoted by vertical green arrows. In most of the time the ‘neurons’ are contending for the right to use the single high-speed bus. (Color figure online)

The lack of possibility of storing information through changing timing led to the need to use a separated "memory" unit, where unique signals (unlike "stored and retrieved directly" (Sterling and Laughlin 2017) in biology) are used to store and retrieve the information. The input and output sections of the model are implemented as numbered storage cells. Given that computing systems are assembled from pre-fabricated functional blocks (Patterson and Hennessy 2017), those sections must be wired to the processing unit. The synaptic weights $W_i$ are stored in those memory cells and accessed through the shared medium of the bus. The shared medium must be made "private" when transferring data. Most of the time is spent with contending for the right of owing the bus, see Fig. 5, and in detail (Végh 2020). This non-payload time is especially disproportional for the neural-mimicking communication, where the neural messages comprise just 1 to 3 bits (Sterling and Laughlin 2017) of information.

Storing information in synapses has two advantages: it is directly wired to the computing unit, and all synaptic weights can be reached quickly and simultaneously (Sterling and Laughlin 2017). In a technological implementation, it would need to store all synaptic weights in processor registers, which not only can be accessed "instantly" (without needing to use bus arbitration), but the processor can perform operations with all of them simultaneously. The sequential operation of processors alone reduces their operating speed by orders of magnitude for many synapses. The distance between storage and computing units makes the transfer time orders of magnitude higher (Végh 2020) and, in this way, surely not negligible apart from computing time. The temporal behavior of communication also reduces the achievable performance by orders of magnitude (Végh 2020). Unlike in the parallel bus system of biology, only one communication action can use the shared medium at a time, and a considerable offset time must be used for contending for the private use of the shared medium.

**Fig. 6:**
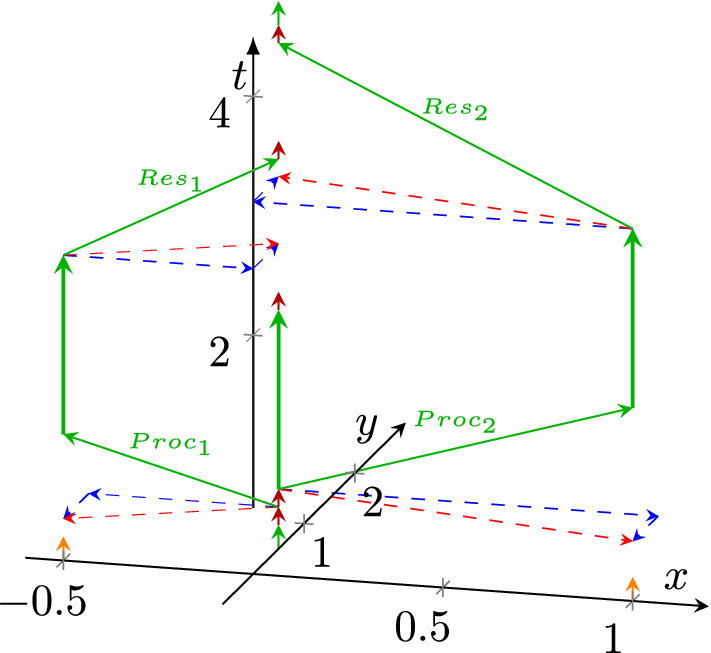
The temporal operating diagram of a parallelized sequential (distributed) computing system: one processor coordinates the work of fellow processors, causing an inherent efficiency bound

The temporal behavior of the "parallel computing" (or distributed computing; a shorthand notation for the parallelized sequential processing) is shown in Fig. 6. The figure shows that distributing parallelizable task fragments and collecting their part results, initializing the tasks, and summarizing part results takes time. Because of the principle of how processing is organized, these activities contribute to sequential (non-parallelizable) and/or parallelized parts of the processing; furthermore, the need to cooperate may introduce more idle waiting. As is shown by the tendency of having assistant cores in the most successful supercomputers on the TOP500, reducing the sequential-only portion of the task increases parallelization gain.

**Fig. 7:**
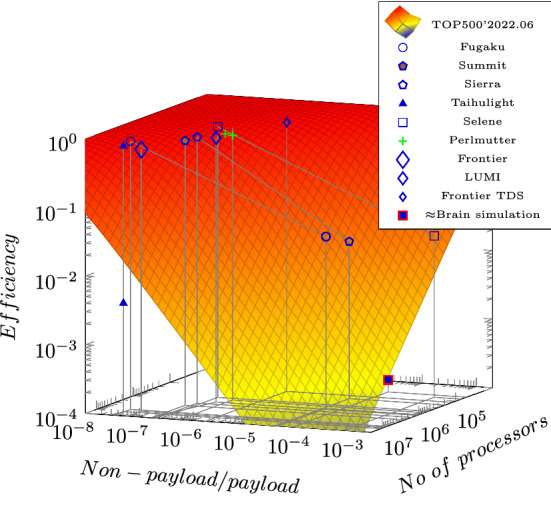
The temporal behavior of the technological components results in that the *payload* efficiency of vast computing systems sharply decreases as the number of processors increases; the architecture defines the parallelization efficiency. Notice the reasoned guess for the efficacy of simulating the brain, resulting from the vast numbers of computing units and the disruptive workload

As analyzed in Végh (2021), these temporal effects reduce computing efficiency for vast computing systems to a drastically low level, see Fig. 7. Two classic ways to benchmark efficiency are to run the same standard benchmark programs on widely different architectures and to run various real-life programs on the same computer architecture.

The decreasing contribution of the interconnection made it evident that most "real-life" programs (programs solving real problems) and the benchmark program High Performance Linpack (HPL) have different efficiencies, forcing researchers to introduce a new benchmark program High Performance Conjugate Gradients (HPCG). (Given that HPCG needs iteration, and the organizer core needs to send/receive parameters multiple times, it is used to imitate "real-life" programs.) That is, it was discovered that the workload affects the computing efficiency of the system. As analyzed in Végh (2020), various contributions to the inherent idle time (sometimes also called "parallelization delay") influence the achievable parallelization gain, and the workload (the type of computing) is one of those contributions. The theoretical efficiency derived by the time-aware computing surface shown in Fig. 7 is replaced by an "empirical" efficiency in the non-time-aware computing (without theoretical underpinning). The strong dependency of efficiency on the number of processors is not understood (although experienced): in vast systems, the benchmark HPCG uses only a fragment (about 10%) of the total available cores. Using more cores decreases the efficiency of the system.

Latterly, one can precisely measure the speedup for different applications running on High Performance Computing (HPC) systems, changing both the number of cores and the workload (using various applications). The careful analysis (D’Angelo and Rampone 2014) of results of running bioinformatics applications pointed out that the speedup curve has a maximum and breaks down for a higher number of processor cores: "*The execution time and the speedup on IPDATA reach the best values within about 90 processors*. Furthermore, that ..."*the parallel version is up to 30 times faster than the serial one*".

The effect itself was discovered early: "*there comes the point when using more Processing Units (PUs) ...actually increases the execution time rather than reducing it*" (Singh et al. 1993). In that paper (at a different workload and architecture) the achievable parallelization gain was about 8, and it was achieved using 20–30 processors. The old experience, despite the vast improvement in parallelization technology, returned in a technologically different form: for those applications, the need for communication defines the achievable speedup. The theoretical interpretation was given in Végh (2020, 2021), furthermore a correct model of computing is suggested in Végh (2021).

Unfortunately, as discussed in Végh (2021), also neural networks, including those used for deep learning, can be used with reasonable efficiency only at a "toy level" or slightly above it (although, at their size, they can perform a valuable job, as discussed in D’Angelo and Palmieri (2021)). The efficiency decays sharply as the problem (and network) size increases. Per our theoretical expectations, among others, that computing and communication can mutually block each other (Végh 2021), it was found experimentally in connection with training neural networks (Keuper and Pfreundt 2016) that:

- *"strong scaling is stalling after only a few dozen nodes"*
- *"The scalability stalls when the compute times drop below the communication times, leaving compute units idle. Hence becoming a communication bound problem."*
- *"the network layout has a large impact on the crucial communication/compute ratio: shallow networks with many neurons per layer ...scale worse than deep networks with less neurons."*

In addition to these effects, the Single Processor Approach (SPA) (Amdahl 1967) requires using Input/Output (I/O) instructions (and non-payload time to organize communication), raising the ratio of the non-payload portion (Végh 2021) drastically. This is why, especially for neural simulation, admitted that: "*artificial intelligence, ...it’s the most disruptive workload from an I/O pattern perspective*."^7^ This is why a growing portion of supercomputers reduce their number of processors when running heavier workloads. Benchmarks HPCG decreases the achievable efficiency by order(s) of magnitude (and the top efficiency can be achieved using an order of magnitude fewer processors), and the AI workload makes it impossible to run an application with reasonable efficiency.

Besides, the analog computation performed by the membrane is imitated digitally by integrating the received charge in short periods (grid time), which makes the "quantal nature of computing time" visible (Végh 2019). This is why both special-purpose hardware (HW) brain simulators and software (SW) simulators running on general-purpose supercomputers can simulate *the operation (not to be confused with filling up the equivalent memory capacity with data* (Kunkel 2014)) only a tiny fraction of our brain’s operation (van Albada 2018). These technological nuances cause that "*any studies on processes like plasticity, learning, and development exhibited over hours and days of biological time are outside our reach"* (van Albada 2018).

The serial operation adds one more issue. The software repeats adding charge contributions in a cycle without being aware at what time that contribution arrived and what is the relation of the partial sum of potential contributions to that of the membrane potential. In this way, the time of firing can happen only after finishing the cycle, which on one side smears the time of firing over the operation of the cycle. On the other side, it makes it hard to interpret Hebb’s observation, given that all contributions (inputs at the synapses) arrive at the "same time". Because of this difference, for changing synaptic weights, special mathematical procedures (such as gradient descent) had to be introduced. They introduce their specific issues (such as vanishing gradient or the need to calculate the gradient of noisy signals) and distribute synaptic learning to "foreign" synapses. Handling the synapses grouped in vector or tensor means that the effect of learning, in a single synapse, experienced in the many-parameter space, is shared between all participating synapses, whether they contributed or not to learning. This latter effect dramatically contributes to the experienced unreasonably long training times and over-fitting (Bengio et al. 2016); accompanied by the lack of explicit time parameters and misfitting.

One of the significant features of technological computing systems is that neither the computing time (central clock) of their processing units nor the transmission speed (of the electromagnetic waves) between them can be changed. Correspondingly, the learning mechanisms (even in the so-called neuromorphic systems) must be drastically different. The lack of time in technological computing does not enable implementing learning mechanisms similar to those known in biology, and machine learning requires introducing "training" or "test" mode switches. For a review see Schuman et al. (2017). The other significant point is that technological computing lives in a world of "instant transmission", i.e., only logical sequencing exists, but no temporal sequencing. When analyzing algorithms, the "time of computing" is included instead of the correct "effective computing time", including transfer time. This difference results in unrealistic estimations in the scaling of algorithms. As was found in D’Angelo and Rampone (2014), "*the memory and the execution time required by the running are of O(n3) and O(n5) order*". In other words, when the problem size increases by a factor of $10^1$, the memory demand rises by a factor of $10^3$, while the execution time increases by a factor of $10^5$; that is *the efficiency of computing decreases by a factor of* $10^2$.

The issue mentioned in connection with Fig. 2, the idle time, leads to further troubles in the case of more complex systems. The gates marked by red circles in Fig. 2 are directly wired. However, when transferring large amounts of data needed for the operation, for example, elements of a vector or a matrix, on a finite width bus, the data must be serialized at the sender and deserialized at the receiver. A typical example is that deep learning figures show *logical wiring*. Still, the *physical wiring* involves also some bus, so the real temporal behavior of neural messages is as shown in Fig. 5. The effect was pointed out both experimentally (Keuper and Pfreundt 2016) and theoretically (Végh 2021), and is a major reason why AI development stalled (Hutson 2020). As the model requires, the computation (with a vector or a matrix) cannot start until the last element is delivered to its corresponding input section. This requirement automatically increases the transfer time by many folds as many elements are considered, and correspondingly, it increases the idle time and decreases efficiency.

However, one may to *not* consider the needed synchronization (it is easy to do so, given that no auto-synchronization occurs) and start the calculation as soon as the overall computing process begins. Analogously to the case of a one-bit adder, some results will always be available in the output section of the tensor. After all, inputs arrive at the input section of the tensor, and the corresponding computation is performed, the result of the output section will match the result expected based on the mathematics. Before that time, we can read out the output section, but it does not have much sense: we can be sure we have the correct result only when the computation in the above sense is completed. *Before that time, the result may or may not be valid.* The faster the tensor unit computes, the worse.

A popular and frequently occurring idea is to use memristors for storing synaptic information (Chicca and Indiveri 2020; Strukov et al. 2019). It sounds good that "*The analog memristor array is effectively the neural network laid out in the form of a crossbar, which can perform the entire operation* ***in one clock cycle***" (Kendall and Kumar 2020). In brackets, however, fairly added, that "*(not counting the clock cycles that may be required to fetch and store the input and output data)*". Yes, all operands of the memristor array must be transferred to its input (and previously, they must be produced), and the results must be transferred to their destination. The total time of the memristor-related operations shall be compared to the total time of conventional operations to make a fair comparison.

The case is even more complicated when introducing different buffers and time stamping. Time stamping preserves the correct biological time. Still, messages may arrive at a physical time when the computing process has already performed the calculation of the state at that biological time, in the absence of the data still waiting somewhere in the queue. A signal coming from a physically distant position of an overloaded system has a good chance of being delayed in one buffer. It arrives at the destination with considerable delay to its expected arrival time. One possible handling is that "spikes are processed as they come in and *are dropped if the receiving process is busy over several delivery cycles*" (van Albada 2018). This handling is believed to enhance the efficiency of the system. Given that the physically distant artificial neurons will have a longer delivery time, they will go to the end of the queue of the input events. Because they are at the end, they have good chances of being dropped (in biology, distant neurons are often controlling local neural assemblies (Buzsáki and Wang 2012); so in their technological implementation, the remote control will be missing).

If they are not dropped because of their late technological delivery, they will arrive at a simulated time that has already passed. Given that the simulated time of their technological processing is already gone, the two bad options are to drop them after investigating their time stamp or, without examining it, process their message content at the wrong time. An extraordinary chance to mishandle those events, that some delayed event will survive in their buffer the end signal of the experiment, and after terminating the investigation (providing no more inputs), they will arrive as valid events (with the correct timestamp from the past), providing the sensation that "Lack of Sleep Could Be a Problem for AIs".^8^

In vast artificial neural systems, "*Yet the task of training such networks remains a challenging optimization problem. Several related issues arise. Very long training time (several weeks on modern computers, for some problems), the potential for over-fitting (whereby the learned function is too specific to the training data and generalizes poorly to unseen data), and more technologically, the vanishing gradient problem*" (Bengio et al. 2016). In the light of our analysis, we can make a substantial addition. Given that the *time* (in its many manifestations) is not present in the many-parameter fitting in explicit form, its effect is attributed to some synaptic weight(s), resulting in misfitting.

One can expect that considering the temporal behavior, in any form, can enhance learning. Some works (for a review see Végh (2021)) already guessed that the technological speed of propagating information may be an issue for artificial networks. A trivial way to provoke faster propagation is to include fewer computing nodes in the network. "*The immediate effect of activating fewer units is that propagating information through the network will be faster, both at training as well as at test time.* Bengio et al. (2016)" However, a natural consequence is that (see their Fig. 5): "*As *$\lambda _s$ *increases, the running time decreases but so does performance.* (where $\lambda _s$ can be understood as a trade-off parameter between prediction accuracy and parsimony of computation)"

## The Effect of Periodic Operation

In biology, the observers start their observation "in medias res": in a living organism, where a stationary, stable "parameter space" exists. However, learning may change the pre-wired settings (McKenzie et al. 2021), both in the synaptic strengths and the number of participating network nodes. Studying learning (especially pre-wired states) needs extreme caution and is challenging not only from a technological point of view.

In technology, the experimenter must set up synaptic weights, connections, and other factors influencing neural operation, requiring pre-wiring of the system. Usually, the system is allowed to learn (i.e., set its weights according to the rules) from an initially random or uniform or some otherwise pre-set state. The lack of information about the initial parameter space settings, and the need to produce a valid initial space setting for the beginning of the training, needs to make a bargain. Given that no synchronization signal is provided, the system assumes all signals to be valid, including the additional feedback, recurrent relations, and other signals (for a review see Schliebs and Kasabov (2013), Schuman et al. (2017)).

This also means that *the computed feedback may reach the neurons in the previous layer before the previous layer could compute their needed inputs*. Because of the lack of synchronous signaling, the computation considers the feedback signal as a full-value signal, even if it is based on uninitialized signals. The result is that even the weights that were originally correct may be destroyed, and the starting point moved in the parameter space to a wrong position, even if initially the point was at the right position (Végh 2021). This effect is topped by the difference that biology naturally implements a limitation for the value and the speed of change for its biological weights (biological signals, including signatures of neural activity, are non-stationary and non-linear), while the "need for speed" in technological computing does not implement such moderation.

The role of time in learning comes to the light demonstratively when analyzing video recordings. This corresponds to the training based on a series of slightly different samples, where various objects vary with varying speeds from frame to frame. One time constraint is how frequently the sample object changes and how quickly its analysis is performed. One can expect that a slow change gives more time for the system to learn; the rapid changes cannot be learned: seen too few times. Our expectation on the role of time (mismatching) is confirmed directly via making investigations in the time domain. "*The convolutional neural networks models are more sensitive to low-frequency channels than high-frequency channels*" (Xu 2020): the feedback can follow the slow changes with less difficulty than the faster changes.

## Conclusions

The time needed to transfer information in technological computing is mostly ignored and is not always taken into account in biological computing. The computing model, proposed by von Neumann, correctly describes both kinds of computing, but the unacceptable simplifications lead to failures. The different implementations lead to drastically different features in computing, which makes true biology-mimicking simulations impractical when exceeding toy-level complexity. Novel technologies could be developed to fabricate more appropriate biology-mimicking computing systems. But first, it must be accepted that in the two implementations, the speed of information transfer differs by several million times, and this difference leads to conceptual errors in designs and explains the failure of vast biology-mimicking systems. The temporal dimension, crucial for biological neural operation, cannot be appropriately handled in current technological imitations of neuronal networks.

## Data Availability

N/A.

## Footnotes

1 It provides a ’Begin computing’ signal (Végh 2021)

2 In this discussion, we use the model that the membrane is a simple (voltage-controlled) condenser. It is known, however, that there are "transients" (Stemmler and Koch 1999), [30] in the voltage of the membrane: the effect of the finite conduction speed is visible even within the membrane of the neuron

3 Given that the power consumption depends mainly on the "flops", these unintended changes are primarily responsible for the excessive power consumption. A one-bit adder may make up to 1.4 times more flops than strictly needed. A 64-bit adder may make up to $1.4^{64}$ unneeded flops.

4 This feature can easily be misidentified as memristance (Strukov et al. 2008) in the time-unaware model. Five decades ago, even *memristance* has been introduced as a fundamental electrical component, meaning that the electrical resistance of the memristor is not constant but depends on the history of current that had previously flowed through the device (Strukov et al. 2008). Handling the "history" actually means time-aware handling. There are, however, some serious doubts as to whether a genuine memristor can exist in physical reality (Abraham 2018). In the light of our analysis, some temporal behavior exists; the question is how much it is related to material or biological features if our time-aware computing method is followed.

5 An interesting historical parallel that computer EDVAC used delay lines with *msec* processing time for information storage

6 The primary goal of that investigation was to prove that learning to some measure is "pre-wired"

7 https://www.nextplatform.com/2019/10/30/cray-revamps-clusterstor-for-the-exascale-era/

8 https://www.lanl.gov/discover/science-columns/top-columns-and-blogs/2020/sciam-artificial-sleep.php

## Code Availability

N/A.
